# A Review on Regulation of Irrigation Management on Wheat Physiology, Grain Yield, and Quality

**DOI:** 10.3390/plants12040692

**Published:** 2023-02-04

**Authors:** Zhuanyun Si, Anzhen Qin, Yueping Liang, Aiwang Duan, Yang Gao

**Affiliations:** Institute of Farmland Irrigation, Chinese Academy of Agricultural Sciences, Xinxiang 453002, China

**Keywords:** wheat, irrigation management, water productivity, physiology, yield and quality

## Abstract

Irrigation has been pivotal in sustaining wheat as a major food crop in the world and is increasingly important as an adaptation response to climate change. In the context of agricultural production responding to climate change, improved irrigation management plays a significant role in increasing water productivity (WP) and maintaining the sustainable development of water resources. Considering that wheat is a major crop cultivated in arid and semi-arid regions, which consumes high amounts of irrigation water, developing wheat irrigation management with high efficiency is urgently required. Both irrigation scheduling and irrigation methods intricately influence wheat physiology, affect plant growth and development, and regulate grain yield and quality. In this frame, this review aims to provide a critical analysis of the regulation mechanism of irrigation management on wheat physiology, plant growth and yield formation, and grain quality. Considering the key traits involved in wheat water uptake and utilization efficiency, we suggest a series of future perspectives that could enhance the irrigation efficiency of wheat.

## 1. Introduction

Wheat (*Triticum aestivum* L.) is one of the major crops and occupies an essential position in agricultural production, providing around 20% of calories and protein in the human diet [1]. Global wheat production is approximately 761 Mt in 2020 [1]. In order to meet the expected global grain demand by 2050, wheat production must be improved continuously in the context of climate change [2]. Van Dijk et al. [3] suggested that grain production should be increased by 35–56% to meet global food demand by 2050, by 30–62% when accounting for climate change. Water shortages, low precipitation, and drought stress occur regularly during wheat growing periods in arid and semi-arid regions which affect wheat performance through the reduction of plant growth parameters and disturbance of the crop water relations, limit the development of root system, alter physiological processes such as photosynthesis and respiration [4] and ultimately, affect wheat production, grain quality, and water productivity (WP) [5].

Water deficit is widely reported for global wheat production. In the North China Plain (NCP) of China, the Texas High Plains of USA, and the semiarid regions of Iran, water scarcity is the main constraint influencing wheat production [6,7,8]. Irrigation is an important agronomic practice to meet the normal demand for wheat production, especially in arid and semiarid regions. At present, water resources available for irrigation are very limited and how to irrigate the limited water to obtain the most benefit per unit of water is a great important issue. It is necessary of developing water-saving irrigation theories and technologies to maintain sustainable wheat production and improve WP [9]. In this paper, we reviewed the advance in the regulation of irrigation methods and irrigation management on wheat physiological properties, yield and components and grain quality.

## 2. Effects of Irrigation Management on Wheat Physiology

With the increasing in water shortage, several water-saving irrigation patterns in winter wheat have been practiced widely, different irrigation methods and irrigation scheduling regulate wheat physiology in different aspects which cause different impacts on wheat growth and water utilization. There is an introduced focus on the effects of different irrigation patterns on the regulation of wheat physiological as follows.

### 2.1. Deficit Irrigation

Deficit irrigation is an important water-saving practice in irrigated agriculture. Deficit irrigation technology can save water resources while maintaining or even improving yield by balancing the relationship between reproductive and vegetative growth [9,10]. Appropriate irrigation scheduling of deficit irrigation is important to promote crop yield and WP. Regulating the amount and frequencies of irrigation water and irrigation during periods of greatest crop demand all have the potential to reduce water losses while maintaining or improving yield [11]. The efficacy of irrigation scheduling in deficit irrigation depends on the irrigation amount [12]. Some studies reported that the increase in irrigation amount increased the leaf water potential, stomatal conductance (Gs) and photosynthesis [13], but large irrigation amount with long irrigation intervals produce an alternation of excess water supply and water deficit stress, which increases Gs and transpiration for a short time right after irrigation and reduces leaf water potential and photosynthesis before the next irrigation [14]. However, increasing irrigation frequency changes the spatial distribution of soil water, soil water storage, and soil temperature, promotes root growth [15], regulates stomatal movement, and reduces transpiration, then improves grain and WP [16]. In addition, several studies have stated that with the same irrigation amount, different irrigation frequencies produced different impacts on the physiological processes of wheat. Chen et al. [17] mentioned that 7 d irrigation intervals led to a higher wheat grain yield than 13 d and 10 d irrigation intervals because of increased wheat root length and root weight significantly, resulting in a change in photosynthetic capacity. While Jana et al. [18] suggested that when receiving a similar amount of water, compared with irrigation once a week, irrigation three times a week attained a high wheat yield because of a higher maximum quantum yield of photosystem II (Fv/Fm). The different conclusions may be explained by the difference in soil properties and irrigation volume.

Irrigation during periods of high wheat demand for water has significant effects on wheat growth, grain yield, and WP. The soil water status at different growth stages have different effects on the photosynthetic, physiological characteristics, and grain yield [19,20]. Cao et al. [21] showed that winter wheat irrigated at the heading and grain-filling stages have a higher grain yield because of the largest leaf vitro rate of water loss, Gs and leaf water potential. Xue et al. [22] reported that irrigation between jointing and anthesis periods significantly increased wheat yield by increasing photosynthesis and remobilizing pre-anthesis carbon reserves. Supplemental irrigation during critical stages of crop development has been used to save water and maintain or improve grain yield in rainfed wheat [10,16]. Tadayon et al. [23] found that supplemental irrigation at the stem elongation stage of rainfed wheat can obtain the highest grain yield, which is attributed to a higher photosynthetic and transpiration rate, Gs and substomatal CO_2_ concentration. Ali et al. [24] showed that the photosynthesis, chlorophyll content, and WP of winter wheat significantly increased with the increase in supplemental irrigation at the jointing, flowering, and grain-filling growth stages under the limited water resources condition. In addition, it has been reported that the application of supplemental irrigation at flowering and grain filling stages with deficit irrigation 75% saved irrigation water and resulted in a lower reduction of grain yield compared with 100% of irrigation through maintaining chlorophyll content and Gs [25].

### 2.2. Regulated Deficit Irrigation

Regulated deficit irrigation is an effective irrigation management technology when irrigation water is scarce. Its core involves in the adjustment of irrigation water based on the phenological period and physiological characteristics of crops [26]. Hence, it is important to understand the water stress responses and physiological mechanisms for water stress resistance of wheat during different growth periods. Several researchers found that winter wheat was highly resistant to moderate water stress before jointing and many negative effects of water stress can be eliminated after rehydration, such as photosynthesis and transpiration rates can quickly recover, or even exceed the values before water stress [27]. Xue et al. [22] found that short-term drought at different stages after the jointing stage could reduce wheat yield because of reduced photosynthetic rate and lead to an increase in catalase and peroxidase activities and an increase in malondialdehyde and proline contents. Wu et al. [28] stated that water scarcity during flowering and grain-filling stages can inhibit and reduce photosynthesis and increase leaf senescence, which may diminish the contribution of pre-anthesis assimilates to yield. Some researchers found that no irrigation at the reviving and jointing stages could improve leaf photosynthetic traits by increasing Gs, Fv/Fm, and the effective quantum yield of PS II (Φ_PSII_), thus increasing grain yield [21].

To realize the goal of attaining high yield and saving water of regulated deficit irrigation, it is needed not only for the appropriate irrigation period but also a grasp of the degree of water deficit. Some scholars showed that the transpiration rate was very sensitive to soil water deficit and in a mild water stress condition, the transpiration rate decreased with increasing water deficit while the photosynthesis rate remained unchanged [29], ultimately, improving WP without significantly impacting photosynthesis and yield [30]. Moreover, Kang et al. [10] also reported that spring wheat receiving reduced irrigation water by about 20% during early vegetative stages produced a grain yield equal to or greater than the wheat that was fully irrigated. This is a result of regulating the water deficit at the appropriate time to significantly inhibit the transpiration rate without decreasing the photosynthetic rate significantly, ultimately, WP is increased.

In addition, some studies have also shown that regulated deficit irrigation can change stomatal opening by controlling the growth of plant roots and then affecting leaf water potential. When the soil water is scarce, the wheat root tip responded rapidly to the change of soil moisture to synthesize ABA and transported it through the xylem to the leaves by transpiration stream, then regulated stomatal opening to control the gas exchange between plants and the atmosphere, then reduced the water consumption of wheat. As a result, WP can be improved [31,32].

### 2.3. Alternate Furrow Irrigation

Furrow irrigation has been shown to improve the efficiency of irrigation by planting crops by furrow and ridge in a field [33]. Several researchers showed that compared with traditional irrigation, furrow irrigation saved water and increased yield due to higher soil water content and soil temperature [34,35], which increased leaf area index, photosynthesis rate, Gs, dry matter accumulation and yield [36,37]. Ali et al. [38] also showed that furrow-irrigation significantly improved soil water content to increase the chlorophyll fluorescence parameters, such as quantum yield of PSII (Φ_PSII_), electron transport rate (ETR), the performance index of photosynthetic PSII (Fv/Fm) and transformation energy potential of PSII (Fv/Fo) which were significantly positively associated with the photosynthesis, biomass, and yield production.

Alternate furrow irrigation as an approach to partial root-zone drying irrigation is applied through crops planted on ridges and water is applied in furrows to wet only part of the bed. Alternate furrow irrigation requires parts of the root zone to be dried and wetted alternately [10] and water uptake from the wet side of the root system to maintain a favorable plant water status for meeting crop normal growth, while the part of the root system in dry side generates a root-sourced signal (ABA) is transmitted to the shoot, where it inhibits the stomata opening so that water loss is reduced and WP is improved [39]. Another mechanism for improving WP is to promote root growth, enhance water uptake capacity and decrease water loss. For example, Du et al. [40] showed that in comparison with conventional irrigation, alternate furrow irrigation increased root length density, decreased water potentials and Gs, reduced growth redundancy, changed the distribution of carbon assimilates, and ultimately, increased production [10].

Numerous results indicated that if the Gs was properly reduced, the changing trend of photosynthesis was inconsistent with transpiration in alternate furrow irrigation. For example, Mehrabi and Sepaskhah [36] showed that compared to traditional irrigation, alternate furrow irrigation achieved an optimum yield with saving water resulting in Gs decreased significantly, along with significantly reduced water loss via leaf transpiration but not significantly reduced photosynthetic rate.

### 2.4. Drip Irrigation

Drip irrigation is recognized as a high-efficient water-saving irrigation technology that has been widely used in arid and semi-arid areas. It has been gradually adopted for winter wheat production not only due to improve yield and quality but also to increase WP [41]. Drip irrigation uses plastic tubing to drip water to crop root zones and avoids water loss in non-root zones [42]. Drip irrigation affects the distribution of soil water and soil air permeability [43]. The changing of the root-zone soil environment affects the root morphological growth and root water uptake patterns in the soil profile which is very important for crop growth, photosynthesis, and grain production obtaining [15]. Previous reports showed that compared with traditional irrigation, there was a higher root length density of 80 cm below the soil surface in drip irrigation, which promoted the absorption and utilization of water in deep soil layers [42]. In addition, optimizing irrigation amount and frequency of drip irrigation can maintain higher soil water content in the topsoil layers where the main part of root distribution for winter wheat [44]. However, Liu et al. [12] stated that excess irrigation frequencies create sustained wetting fronts which leads to constraining the supplies of oxygen for the root, inhibits root growth, then limits photosynthesis, yield formation, and water uptake. Camposeo et al. [45] concluded that with the decrease in irrigation frequency, the root length density along the soil profile decreased by more than 76%.

Subsurface drip irrigation (SDI) is an irrigation technology developed from surface drip irrigation and the drip emitters are placed in a plow layer to directly pour water into the root zone for the growth of crops. SDI has better and more stable soil water conditions in the middle and deep soil layers. Romero et al. [46] indicated that SDI produced a larger horizontal distribution of fine roots in the soil profile and stimulated a deeper root development than surface drip irrigation. As well, SDI was found to manipulate rooting depth to make full use of soil water storage [42,43], improved leaf water status, increased photosynthetic rate, maintained transpiration rate [47] and increased light energy utilization efficiency [48], ultimately, increased yield and WP.

Drip fertigation is highly efficient because of the direct application of water and nitrogen within the rooting zone at very low rates, which alters the distribution of water and nitrogen in the soil and changes the physiological and biochemical characteristics of roots [42]. The change in the soil environment in the root zone generates the root-sourced signal (ABA) [31], then affect the shoot through the root system [49], such as improving wheat photosynthesis [50], biomass production [51], ultimately, it will have a positive role in the yield and water-nitrogen use efficiency.

## 3. Effects of Irrigation Management on Wheat Growth and Yield

### 3.1. Effects of Irrigation Method on Wheat Growth and Yield

**Figure 1:**
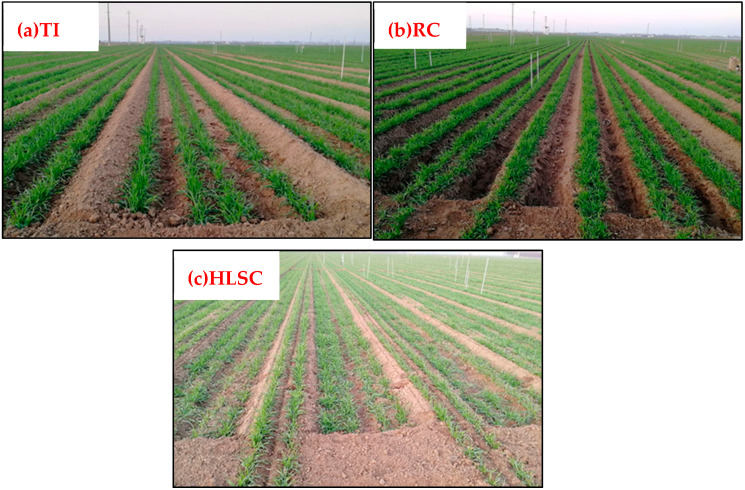
Field schematic diagram of different surface irrigation methods. (**a**) Traditional irrigation (TI), (**b**) Raised bed cultivation (RC), (**c**) High-low seedbed cultivation (HLSC). From Si, ZY. 2020. Effects of different cultivation methods on growth and water-nitrogen use efficiency of winter wheat (Doctoral Dissertation). Chinese Academy of Agricultural Sciences [54].

In many areas around the world, rainfall cannot meet the water demand during the wheat growth period and supplementary irrigation is necessary to maintain wheat production [52]. At present, traditional surface irrigation (TI) is an important irrigation from around the world because of its advantages of simple field facility and easy implementation (Figure 1). For example, surface irrigation accounts for more than 85% of the total irrigated area for winter wheat in North China. Traditionally, farmers build borders in the field and irrigate the field along the border or use hoses to assist irrigation [53].

Raised bed cultivation (RC) is a planting method in which beds are raised in the field, crops are planted on beds and irrigation is carried out in furrows [55]. RC pattern has been applied to irrigated and dryland farming areas in many countries [53,56]. Studies have shown that RC can effectively improve farmland micro-environment, improve soil physical structure, reduce irrigation quota, and improve WP and nitrogen use efficiency [53,57]. Ahmadi et al. [58] revealed that full irrigation resulted in thicker and thinner roots in the shallow soil depths of the raised bed and flat planting systems, respectively. In contrast, under deficit irrigation and rainfed conditions, thinner and thicker roots were respectively developed in the shallow soil depths of the raised bed and flat planting system. Rady et al. [59] found that beds combined with irrigation water at 100% crop evapotranspiration (ET_c_) followed by 80% ET_c_ significantly increased growth characteristics, grain yield, and its components compared with TI combined with irrigation water at 60% ET_c_. Devkota et al. [60] conducted experiments in Uzbekistan and showed wheat produced 12% higher grain yields and 27% higher WP, while 11% less water was applied, under RC than under TI. A study in Egypt showed that the adoption of RC led to a 937 kg ha^−1^ (12.79%) increase in yield, an 824.63 m^3^ ha^−1^ (15.05%) reduction in irrigation water application, 5.56% increase in WP and an 11.80% reduction in downside yield risk [56].

In semiarid and arid regions where precipitation is scarce while evapotranspiration is very large, the ridge and furrow rainwater harvesting (RFRH) planting pattern, has been recently developed by combining plastic-film mulching with ridge-furrow planting. Compared with TI, RFRH significantly increased the soil water storage in the early growth stage and required 50% less irrigation water but it increased the grain yield by 3.3%, 2.4%, and 2.8% with one application in dry, normal, and wet years, respectively [61]. Liu et al. [62] reported that RFRH prolonged the duration of the jointing–anthesis stages and thereby increasing the kernel number in dry semi-humid areas. In the Yangtze River Plain in China, the RC pattern significantly increased soil water drainage, promoted the wheat seedling establishment and root growth, accelerated stem and tiller development, and delayed late-season root and leaf senescence, resulting in higher grain yields [63]. Du et al. [64] also showed that the higher wheat productivity under RC was due to the increased canopy leaf area index (LAI) and light interception benefited from the improved early-season growth and delayed late-season leaf senescence, which compensated for the reduced cropping area under RC caused by the furrows.

The High-Low Seedbeds Cultivation (HLSC) pattern was developed by the Binzhou Academy of Agricultural Sciences, Shandong Province, China, aiming to solve the problems of low land utilization rate and light loss in the TI pattern [65]. In the HLSC, the land is integrated into a plane alternating between high and low beds, and wheat is planted on both beds. During irrigation, the higher beds act as the border and the irrigation is carried out only in the lower bed. Compared with RC and TI, the HLSC significantly increased LAI and aboveground biomass of wheat, mainly attributed to the increased effective spike number. The grain yield of HLSC increased by 22.63% and 27.37% higher than that of TI and RC, respectively. Furthermore, the WP of HLSC was 7.69% and 6.8% higher than that of TI and RC respectively [66]. In the study of Wu et al. [65], two cultivars were used to investigate how HLSC mode increased grain yield and lodging resistance compared with TI. The results showed that the grain yield increase was mainly due to the enhanced spike number per area, while the lodging resistance was improved through the lowered plant height and the center of gravity height in the HLSC model.

In recent years, with the higher requirements for water-saving, efficient, and sustainable agricultural development, drip irrigation (DI), micro-sprinkling irrigation (MSI), and other water-saving irrigation methods were gradually adopted in cereal crop production to relieve severe water shortages. DI and MSI can supply water and fertilizer to root areas at very low rates, which can help promote wheat absorption of water and nutrients [15]. Li et al. [42] reported that the grain yield of DI and MSI was improved by 9.79% and 14.1%, WP of DI and MSI, increased by 12.3% and 17.7% and NUE of DI and MSI, increased by 9.77% and 14.0%, respectively compared with those of TI across the 3 years. Bai et al. [67] compared the average grain yield and partial factor productivity (PFP) under the local surface irrigation regime and found that drip fertigation of 70% of the local recommended fertilizer dose resulted in a yield increase by 4.0% and PFP increase by 48.5%. Fang et al. [44] reported that two irrigation applications (45 mm/application) in a season conducted using DI significantly increased the yield and WP, compared with TI using one single irrigation application at 90 mm/season. Mehmood et al. [68] found that drip irrigation at 60% Field capacity (FC), compared with sprinkler and flood irrigation at the same schedule level, improved grain yield by 5–8% and raised WP by about 1–8% for winter wheat. Zhai et al. [69] reported that MSI either stabilized or significantly increased the grain yield, while reducing irrigation water volumes by 20–40% compared to TI, regardless of the rainfall pattern. In the NCP, lysimeter experiments were conducted under different irrigation treatments to account for ET in the selection of a suitable irrigation method. Subsurface drip irrigation reduced ET by 26% compared to flood irrigation and 15% compared to surface drip irrigation, with significantly higher grain yield and biomass formation due to decreased evaporation losses [70]. Sidhu et al. [71] found that irrigation water savings were 48–53% in rice and 42–53% in wheat under a combination of sub-surface drip fertigation and conservation agriculture compared to a flood irrigation system.

Partial Root-zone Drying (PRD) is an effective irrigation method that saves water although it can affect root activity through the heterogeneous distribution of moisture in the soil [72,73]. The PRD technique can be achieved through different irrigation methods, among which we find a drip, furrow, or micro-sprinkler depending on the crop species and soil texture [72]. Ahmad et al. [58] experimented to investigate the effects of two techniques (use of ground covers and PRD) for increasing crop production under limited water resources. They revealed that longer spike lengths, more number of spikelets, and grains were found in full irrigation treatment regardless of ground cover types. While WP and grain nutrient (NPK) contents were more in PRD. Raza et al. [4] conducted a pot experiment in a wirehouse to evaluate the impacts of partial root-zone drying (PRD) and control irrigation on five different wheat genotypes. The results showed that values of growth, physiological and water-related parameters were higher in the control treatment except for leaf water potential, osmotic potential, total sugars, and proline contents. All five wheat varieties showed greater antioxidant enzyme activities in PRD compared with the control treatment. Iqbal et al. [74] found that higher development, physiological and yield-related parameters of wheat were seen in the full water system compared with PRD and deficit watering system. More ABA and osmotic modification were found in PRD-treated plants than in other irrigation systems. Leaf water use efficiency was likewise higher in PRD plants compared with the full water system and deficit watering system.

The spatial distribution of soil water and nitrogen was greatly regulated by different irrigation methods, which then affected the development of wheat roots and the establishment of root architecture and finally affected wheat growth and yield formation. Li et al. [42] reported that the root length density (RLD) of TI in the 0–80 cm soil layer was significantly higher than that of micro-irrigation, whereas micro-irrigation had a higher RLD than TI below the 80 cm soil layer, which promoted the absorption and utilization of water and nitrogen in deep soil. Lv et al. [75] found that root growth was most stimulated in the topsoil layer and inhibited in the deep layers in the SDI, followed by sprinkler irrigation and border irrigation. Additionally, more fine roots were produced in the BI treatment when the soil water content was low and topsoil bulk density was high. Jha et al. [15] reported the RWU was higher in DI compared to MSI and TI due to the higher RLD in the topsoil under DI. On the other hand, the root water uptake was higher in TI at a deep soil profile below 60 cm, where it had a higher RLD compared to that of MSI and DI. Fang et al. [44] reported increasing irrigation frequency would maintain the topsoil layers with higher soil water contents where RLD was greater which improved crop water use and yield under a limited water supply.

### 3.2. Effects of Irrigation Scheduling on Wheat Growth and Yield

Optimizing the irrigation strategies is another aspect of efficiently utilizing the limited irrigation water. Previous studies showed that the plant height, LAI, aboveground biomass, and yield components (spike number per hectare, kernels per spike, thousand-kernel weight) of winter wheat increased with the increase in irrigation amount [8,41]. However, unreasonable irrigation scheduling will not effectively improve crop yield and instead cause a waste of water resources and a decrease in WP. Traditional surface irrigation is commonly applied three or four times using more than 300 mm of irrigation water during the growing season to obtain a high grain yield [76,77]. This irrigation practice improves grain yield but reduces WP due to supplying too much water [77]. Therefore, an optimal irrigation water management scheme must be developed for ecological security and sustainable development of winter wheat production in this region [78].

Wang et al. [79] showed that compared with the W3 (pre-planting + anthesis irrigation) and W4 (pre-planting + jointing + anthesis irrigation), the W2 (pre-planting + jointing irrigation) increased yield by an average of 7.56–10.58% and 2.06–2.68%, improved WP by 9.95–17.83% and 11.29–22.84%, respectively. Feng et al. [80] reported that the RLD, root surface area density, and root weight density in the 0–0.2 m, 0.6–0.8 m, and 0.8–1.0 m soil layer from T2 (Irrigation at jointing and anthesis) were significantly higher than those from T3 (Irrigation at sowing, jointing, and anthesis) and T4 (Irrigation at pre-wintering, jointing, and anthesis) at anthesis. In summary, irrigation at joining and anthesis that was based on suitable soil water content at sowing increased the absorbing area of roots in both deep and surface soil layers, accelerated the dry matter accumulation after jointing and finally higher grain yield and WP were achieved. Xu et al. [78] investigated how to optimize the timing of two irrigations to improve winter wheat grain yield and found that irrigation at jointing and anthesis optimized crop characteristics with appropriate leaf area index, delayed leaf senescence, extended grain filling duration by 1–3 days, increased biomass post-anthesis and harvest index (HI), then improved grain yield and WP.

The jointing stage is the critical stage of water demand for winter wheat and the occurrence of drought at the jointing stage has a serious impact on plant growth and photosynthesis [81]. On the other hand, irrigation during this stage significantly increases production [82]. Liu et al. [52] reported that supplemental irrigation at the jointing stage significantly increased the amount of N accumulation in shoots at anthesis and pre-anthesis N redistributed to grains and its contribution to grains. Zhang et al. [77] reported that irrigation scheduling of one irrigation application from recovery to jointing for winter wheat could achieve relatively stable yield and higher WP through the 28 growing seasons and should be taken as optimized irrigation scheduling under limited water supply conditions. Fan et al. [20] indicated that delayed irrigation at the jointing stage significantly increased the number of spikes as well as the kernel numbers per spike in wide-precision planting patterns.

Deficit irrigation, defined as the application of water below full crop-water requirements [83], has been promoted in many countries in an attempt to minimize irrigation water use. Water stress advanced the thermal time required from sowing to the maximum aboveground dry matter rate, while the maximum aboveground dry matter accumulation rate and average accumulated rate of aboveground dry matter increased with the increase of irrigation and fertilization regimes [84]. Rathore et al. [85] conducted a 2-year field experiment in a hot, arid environment in Bikaner, India to investigate the effects of irrigation and N application rates on the yield and WP of wheat. The results showed that moderate deficit irrigation (irrigation amount of 80% ET_c_) had the greatest WP and caused a 17% reduction in water consumption with only a 5% reduction in yield compared to full irrigation. Gao et al. [86] conducted a six-year experiment in the NCP to determine whether deficit irrigation combined with reduced N fertilizer rate can mitigate greenhouse gas (GHG) emissions and maintain yield. The result showed that deficit irrigation in wet years and N reduction in normal years and dry years can reduce GHG emissions and maintain yield.

For drip irrigation and other water-saving irrigation technology, Jha et al. [87] found that irrigation methods with suitable irrigation scheduling indeed have the potential to balance the optimal yield and WP. In their study, irrigating six times each with 30 mm of water could achieve the highest yield for drip irrigation and sprinkler irrigation, while irrigating three times each with 60 mm of water gave comparable results for flood irrigation. Mehmood et al. [68] showed that the combination of surface drip irrigation and a scheduled level of 60% FC is reasonable to be recommended for winter wheat irrigation practices regarding better yield sustainability, higher WP, and mitigating N_2_O emission in the NCP. In the study of Si et al. [41], considering comprehensively yield and water-nitrogen use efficiency, the combination of an N rate of 240 kg ha^−1^ and an irrigation quota of 40 mm per irrigation was the optimal pattern for drip-irrigated winter wheat. Dar et al. [88] evaluated the effect of drip irrigation schedules on field water balance, yield, and WP of wheat and demonstrated that irrigating wheat at 15% depletion of FC using the drip irrigation method saves irrigation water in addition to higher grain yield. A two-year field study in the semiarid region of Upper Egypt showed that grain yield and WP were higher by 20% and 59% in the case of I75 (I = 75% of I100) compared to I100 (full irrigation) [89].

Crop systems modeling has been proven to be a useful tool to investigate the impacts of irrigation scheduling on crop productivity and resource use efficiencies of farming systems. Zhang et al. [90] explored optimal irrigation of winter wheat over a 60-year of long-term meteorological data (1961–2020) based on the AquaCrop model in arid and semiarid areas. The simulated results showed that higher irrigation could produce a higher yield, but the incremental yield would be significantly decreased with more irrigation. The optimal irrigation schedules in the wet, normal, and dry years were determined to be first irrigation in the wintering stage with 90 mm and second irrigation in the jointing stage with 0, 30, and 60 mm, respectively. Similarly, Zhao et al. [91] also used the AquaCrop model to optimize irrigation systems over a 35-year of long-term meteorological data (1981–2015) in North China. The results showed that two irrigations at the stem elongation and anthesis stages significantly increased yield, WP, and irrigation water productivity with values of 7.79 t ha^−1^, 1.72 kg m^−3^, and 2.20 kg m^−3^, respectively, compared with those under lower irrigation frequency treatments. In the study of Davarpanah et al. [92], the AquaCrop model was applied to winter wheat in a warm and semi-arid environment in the south-west of Iran to model recommended and probable scenarios of different deficit irrigations under Rotational and On-demand irrigation scheduling. Modeling showed that Rotational irrigation was more adaptable to deficit irrigation practices due to maintaining higher grain yield and WP compared with On-demand irrigation. Indeed, it is possible to maintain grain yield and increase WP by applying deficit irrigation up to 40% of full irrigation under Rotational irrigation. A 28-year field experiment from 1991 to 2018 together with the APSIM mode was used to characterize the yield sustainability of winter wheat under limited-irrigation schemes. The results showed that irrigation before sowing and at the jointing stage (W2) and irrigation before sowing, at the jointing stage and anthesis (W3), can narrow yield gaps, increase wheat yield, and increase the sustainability of crop production in the NCP [93].

Dar et al. [94] evaluated the performance of the CERES-Wheat model for simulating yield and water use under varying soil moisture regimes and indicated that simulated evapotranspiration decreased by 16%, wheat grain yield by 23% and WP by 15% in drip irrigation at 45% depletion from field capacity as compared to drip irrigation at 15% of field capacity. In the study of He et al. [95], the CERES-Wheat model was used to simulate the spring wheat growth in irrigated farmland in Northwest China. The results of the model simulation indicated that if the soil water content is lower than 65% of field capacity in the 1 m depth soil profile during the grain filling and milk ripe stages, the final grain yield could be remarkably reduced even for a short period of water stress. Zeng et al. [96] analyzed 33 irrigation scenarios under different rainfall patterns (wet, normal, and dry years) based on the CERES-Wheat model. The result showed that two irrigation events at the greening and jointing stages alleviated water stress during key winter wheat growth periods, contributing to increasing biomass, yield, WP, and IWP. Kheir et al. [97] used the DSSAT model to simulate a deficit irrigation schedule by different fractions of actual evapotranspiration (ET_c_). The simulated grain yield and water productivity under irrigation based 90% ET_c_ increased by 1.7% and 63%, respectively, compared with farmers’ value in all locations, confirming the importance of irrigation scheduling based 90% ET_c_ in maximizing wheat yield and WP in arid regions.

## 4. Effects of Irrigation Management on Wheat Grain Quality

Wheat is a major cereal crop, whose grains are mainly processed into bread, noodles, and cakes. Different food types made from grains require different wheat qualities. Wheat quality can be classified as strong gluten, medium gluten, and weak gluten varieties according to grain hardness, protein content, and dough stability time [98]. Strong gluten wheat usually has the characteristics of high gluten strength, good extensibility, and long dough stability, which are suitable for making bread [99]. Medium gluten wheat has moderate gluten strength, which is the best material for steamed bread and noodles. With lower protein content and shorter stabilization time, weak gluten wheat is mostly used in making crisp foods such as cakes and biscuits [100]. With the continuous improvement of people’s living standards and the optimization of daily diet structures, a variety of high-quality wheat cultivars and cultivation practices are demanded.

Although China is one of the greatest wheat producers in the world, the country still needs to import large quantities of high-quality wheat due to its noticeable shortage. According to the released data from the National Grain and Oil Information Center, China’s high-quality wheat import volume reached 2.8 million tons in 2020, but it was still far from meeting the rapid growth demand from the food processing industry and consumers [101]. To produce more high-quality wheat, we should not only consider breeding innovative high-quality varieties but also consider adopting optimized farming practices such as water management means [102].

### 4.1. Effect of Irrigation Regimes on Flour Quality and Dough Stability

Soil moisture content has been regarded as one of the most critical factors affecting the quality of wheat grains. In recent years, the increasing demands for medium to strong gluten wheat on the market have made related research for high-quality wheat a hot topic in the world [103]. A great many measures were taken to increase the production of high-quality wheat, including genetic modification, bio-technologies, and agronomic practices [104]. Among them, reasonable water management is shown a more effective way to improve wheat quality at a lower cost, compared to other practices [105].

Previous studies showed that reasonable water management favored the quality of wheat grains. A study conducted in Shandong province indicated that winter wheat should be irrigated 2–3 times during the entire growth period, guaranteeing the formation of wheat’s high quality [106]. It is believed that wheat quality was noticeably improved under the condition of moderate water shortage due to the effects on deepening root growth and increasing dry matter accumulation, which was favorable for producing high-quality grains with acceptable yields [107]. Some studies also reported that increasing irrigation amounts increased wheat yields while decreasing the quality of wheat grains [108]. Some researchers also found that strong gluten wheat had appropriate protein content and flour quality under the condition of moderate drought levels [109].

Varieties × irrigation interactions had significant effects on the tensile properties of wheat grains. Irrigation amounts exceeding 200 mm or less than 110 mm were shown not to be conducive to the improvement of dough properties [110]. With the increasing irrigation amount, dough stability and water absorption also showed a parabolic trend which went increasing at first, reached the peak values under the condition of two irrigation times (applied at wintering and jointing stages), and then decreased with increasing irrigation amount [111]. Moreover, dough stability time and tensile resistance displayed an increasing trend with decreasing irrigation amount [112]. Therefore, proper irrigation amounts were conducive to the improvement of water absorption and dough stability time too. Irrigation during the late growth stage also shortened the dough stability time of strong gluten wheat, bringing lower wheat quality [113]. In contrast, reducing irrigation amount was beneficial to the improvement of dough stability time, but was not favorable to yield formation [98]. Although irrigation > 210 mm significantly shortened the dough stability time, optimizing irrigation regimes was proven beneficial to the improvement of tensile resistance of strong gluten wheat [111]. However, increasing the irrigation amount after anthesis was shown to decrease the extensibility, tensile resistance, and maximum tensile resistance of wheat, whereas reducing the irrigation amount from jointing to the maturity stage of wheat was beneficial to improving dough strength and reducing dough extensibility [114].

### 4.2. Effects of Irrigation Regimes on Wet Gluten Content and Gluten Index

The stability time of wet gluten and dough displayed a parabolic trend with the increase in irrigation amount and the values peaked under the condition of two irrigation events [115]. Irrigation applied after sowing and at jointing and booting stages better-coordinated wheat grain yield and grain number per spike. These irrigation regimes are conducive to both yield and quality and became a basic mode for obtaining high quality and high yield [116]. It was observed that irrigation during the late growing period significantly decreased the protein content and shortened the dough stability time, finally resulting in lower wheat quality [117]. The ratio of glutenin/gliadin, gluten content, and sedimentation value of wheat was significantly increased under moderate drought conditions [113]. The gluten index is an important indicator to reflect gluten strength. Drought in the late growth stage was shown to significantly improve the gluten index of wheat [118]. The unreasonable irrigation regimes might have a significant negative impact on the gluten index, whereas severe drought levels would also reduce the index [116]. Irrigation with a lower limit at 60% field water capacity (FC) at the mid-filling stage of wheat significantly improved the gluten index and dough rheological properties with comparable grain yield, compared to adequate irrigation treatment [119]. Also, adequate soil moisture during wintering was critical to wheat quality. It was shown that wet gluten and dough rheological properties will be improved without irrigation at jointing and flowering stages with enough initial soil water storage during wintering [120].

### 4.3. Effect of Irrigation Regimes on Protein Content and Components

Studies on the regulation effects of irrigation regimes on wheat protein content have long been carried out at home and abroad [121]. The protein content is a reference index for evaluating the high quality of strong gluten wheat [122]. The quality of wheat is closely related to the quality and content of protein [123]. The protein content was significantly and positively correlated with the water absorption of gluten, but high protein content was likely to inhibit the stability time and dough stretching length [124]. Wheat protein components can be divided into albumin, globulin, gliadin, and glutenin. The major difference lay in the order of prolamin and glutenin between the strong and weak gluten cultivars [125]. Reducing irrigation events increased the protein content of high-quality strong gluten wheat, including the content of gluten, albumin, and globulin, while the content of gliadin decreased [126]. Under the treatments with irrigation applied at wintering and jointing stages, the yield and quality of strong gluten wheat could be effectively coordinated [127]. Within zero to two times of irrigation events, the content of albumin, glutelin, and prolamin increased, but the difference in protein contents with an irrigation amount of 140 mm was non-significantly different from that of 200 mm irrigation [128]. The protein content for different strong gluten wheat varieties varied under different irrigation conditions [129]. Some varieties had higher protein content when the total irrigation amount was less than 200 mm, while some varieties had higher protein content when the irrigation amount was >210 mm, indicating that strong gluten wheat varieties responded differently to various irrigation regimes [130].

Previous studies have shown that rainfall was negatively correlated with wheat quality, in which the increase in rainfall events will lead to a decrease in wheat protein content [131]. A field experiment conducted by Wang et al. [132] in Laiyang, North China Plain, showed that the precipitation during the early growth stage of spring wheat was negatively correlated with the protein content and had an extremely significant negative correlation during the middle to late growth stages. Protein content and components in grains had an important impact on wheat quality. The protein content of wheat grains showed a parabolic relationship with the reproductive growth process with valley values appearing at the mid-filling stage and peak values occurring at the flowering and maturity stages [133]. That was probably because the starch synthesis rate of wheat grains was faster than the protein synthesis rate in the reproductive stage [134]. During the period from wheat flowering to the filling stage, gluten content would be reduced when encountering excessive precipitation, thus reducing the content of protein and gluten, resulting in a reduction in gluten elasticity. Under certain fertilization conditions, increasing irrigation exerted a significant dilution effect on wheat flour quality and its protein content and wet gluten content would be reduced with irrigation or rainfall amount increasing [135]. According to the research of Erekul et al. [136], irrigation at the late flowering stage of wheat had a negative impact on wheat flour quality and its protein content and sedimentation values were reduced to varying degrees.

### 4.4. Effect of Irrigation Regimes on Wheat Starch Content

The proportion of amylose and amylopectin played an important role in the taste quality of wheat-related foods [137]. Amylose content was negatively correlated with taste quality, while amylopectin content and swelling power were positively correlated with the quality [138]. When the content of amylopectin was high, wheat flour would have a strong water absorption capacity and high amylopectin content, which was conducive to producing flavorful noodles and bread [139]. Previous studies found that amylopectin was more vulnerable to soil moisture content and reducing irrigation amount appreciably increased amylopectin content [140]. Therefore, suitable irrigation amount was beneficial to starch accumulation and yield formation of wheat [141]. It was also found that the total starch content and amylose content of maize increased with the increases in irrigation times with reasonable irrigation amount, whereas both amylose and amylopectin decreased under either severe drought or water-logging conditions [142]. Removing spring irrigation after jointing effectively improved the amylopectin content and total starch content of strong gluten wheat [143]. Similarly, serious soil water deficit significantly reduced the total starch, amylose, and amylopectin content of wheat, thus effectively increasing the branch/amylose ratio of wheat and the high branch/amylose ratio markedly improved the taste quality of noodles and bread [144]. Under drought conditions, cereal crops had a decrease in the peak viscosity of starch and an increase in the valley viscosity and retrogradation value, thus were improved for their anti-aging performance of starch paste [145]. The crystal and gelatinization properties of wheat starch were significantly affected under drought conditions, thus the quality of wheat starch was impacted [146]. Therefore, it is possible to produce cereal grains with high starch quality through reasonable water management in the agricultural sector [147].

## 5. Perspective for Improving Irrigation Management in Wheat Crops

Understanding the regulating mechanism of the physiological process of wheat under different irrigation regimes will provide the theoretical basis for how to optimize water-saving irrigation technology for sustainable wheat production. The regulating mechanism of irrigation management on wheat physiology, grain yield, and quality needs to be revealed from the following three aspects.

Firstly, the dynamic allocation of limited irrigation water resources should be developed to improve irrigation management and WP. The adjustment of irrigation water is based on the regulation of the water demand of wheat during growth periods, a mild water deficit in vegetative stages causes the transpiration rate to decrease while the photosynthesis rate remains unchanged. Besides, winter wheat is highly resistant to moderate water stress in early vegetative growth periods and many negative effects can be eliminated after rehydration, such as photosynthesis and transpiration rates can quickly recover, or even exceed, which does not affect the accumulation of dry matter in the later periods of wheat.

Secondly, the interaction between roots and soil environments should be investigated under different scenarios of irrigation management. Changing of soil physiological and biochemical characteristics by different irrigation methods and irrigation scheduling stimulate the root to generate a root-sourced signal (ABA) that is transmitted to the shoot to regulate the stomatal opening of leaves, then change the transpiration and photosynthesis rate, physiological water consumption and biomass accumulation. Different irrigation methods and irrigation scheduling change the soil environment of the root zone, affect the root distribution, root morphological growth, and root water uptake patterns in the soil profile, which disturb the utilization of soil water in the different soil layers, thus changing the leaf water status, photosynthetic rate, transpiration rate, ultimately, yield and WP.

Thirdly, the regulation of irrigation management on wheat grain quality should be assessed to balance wheat yield, grain quality, and WP. In the face of climate change and other global challenges, it is of great significance to maintain wheat yield and meet the requirement of high-quality foods by high-efficiency utilization of irrigation water resources. Regulation of irrigation water allocation on starch metabolism is required for understanding grain quality formation. A model of wheat yield, grain quality, and WP is an essential prerequisite for wheat production.

## Data Availability

The data support the findings of this study are available from the corresponding authors upon reasonable request.
